# ROCK Inhibitor-Induced Promotion of Retinal Pigment Epithelial Cell Motility during Wound Healing

**DOI:** 10.1155/2019/9428738

**Published:** 2019-06-19

**Authors:** Hiroyuki Kamao, Atsushi Miki, Junichi Kiryu

**Affiliations:** Department of Ophthalmology, Kawasaki Medical School, 577 Matsushima, Kurashiki, Okayama 701-0114, Japan

## Abstract

**Purpose:**

No standard therapy for RPE tear, a complication of neovascular age-related macular degeneration, exists even though RPE tears cause severe vision loss, and promotion of cell proliferation and/or migration could be a candidate RPE tear therapy. The aim of this study is to evaluate the effect of Rho-associated coiled-coil containing kinase (ROCK) inhibitor Y27632 on retinal pigment epithelial (RPE) cell motility during wound healing.

**Methods:**

Human RPE cells were cultured in media with and without 10 *μ*M Y27632. A luminescent cell viability assay and vinculin immunocytochemistry were used to test the Y27632 effect on RPE cell adhesion. The mean size of vinculin puncta was quantified from immunofluorescence images. RPE cell motility during wound healing was evaluated using time-lapse imaging and measuring cell migration distances and cell coverage rate in wound fields.

**Results:**

The number of adhered RPE and mean size of vinculin puncta were, respectively, 20519 cells and 3.65 *μ*m^2^ under nontreatment and 23569 cells and 0.66 *μ*m^2^ under Y27632 treatment. Cell migration distance and cell coverage percentage for untreated and Y27632-treated cells were 98.9 and 59.4% and 203.4 and 92.5%, respectively.

**Conclusions:**

Inhibition of ROCK signaling by using 10 *μ*M Y27632 promoted RPE cell motility during wound healing by reducing RPE cell adhesion strength.

## 1. Introduction

Retinal pigment epithelial (RPE) tears [1] represent a complication of neovascular age-related macular degeneration (nAMD) [2], a primary cause of blindness in elderly populations in developed countries. RPE tears develop RPE defect areas, leading to photoreceptor cell death and frequently resulting in devastating vision loss [3]. There are no therapies clinically available for patients with RPE tears to date. However, RPE cell defects do not immediately cause irreversible photoreceptor disorder: photoreceptor cells can survive up to 325 days after RPE tear onset [4]. In the clinical course of certain RPE tear cases, resurfacing of RPE defect areas through cell proliferation and/or migration was observed [5–7], and this remodeling preserved retinal function [8].

Cell adhesion is a crucial regulator of cell migration, and vinculin is a core focal adhesion (FA) protein that localizes to cell-extracellular matrix (ECM) adhesion sites [9]. Vinculin activity governs FA protein recruitment and release, thus regulating cell adhesion strength [10]. Structurally, vinculin comprises head, neck, and tail domains; the head domain binds talin [11], the main regulator of integrin activation, and the tail domain binds actin filaments [12]. Notably, vinculin-actin interaction affects cell adhesion: enhancing actomyosin contractility strengthens cell adhesion, whereas inhibiting actomyosin contractility, e.g., by using blebbistatin or Rho-associated coiled-coil containing kinase (ROCK) inhibitor, weakens cell adhesion in a vinculin-dependent manner [13]. Moreover, activated vinculin-mutant cells and vinculin-deficient cells migrate slower and faster, respectively, than wild-type cells [10, 14].

ROCK plays a critical role in actin stress fiber formation [15] and is expressed in ocular tissues, including the corneal epithelium and endothelium, trabecular meshwork, and ciliary muscle, and ROCK inhibitor Y27632 treatment enhances wound healing in corneal epithelial cells [16], corneal endothelial cells [17], and trabecular meshwork cells [18]. Furthermore, Y27632 promotes RPE cell attachment, proliferation, and wound closure [19]. However, studies investigating ROCK inhibitor-induced RPE cell adhesion and motility are limited. In the present study, we investigated whether Y27632 promotes RPE cell migration during wound healing, with the aim being to evaluate Y27632 use as an RPE tear treatment.

## 2. Materials and Methods

### 2.1. Culture of Human RPE Cells

Human fetal RPE cells were purchased from Lonza (Basel, Switzerland) and cultured on dishes coated with CELLstart (GIBCO, Carlsbad, CA, USA) in preconfluent medium (F10 (Sigma-Aldrich Corp., St. Louis, MO, USA) with 10% fetal bovine serum) before reaching confluence and in postconfluent medium (DMEM/F12 (7 : 3) supplemented with B27 (Invitrogen, Carlsbad, CA, USA), 2 mM L-glutamine (Sigma-Aldrich Corp.), 10 ng/mL basic fibroblast growth factor (Wako, Osaka, Japan), and SB431542 (0.5 *μ*M, Sigma-Aldrich Corp.)) after reaching confluence. Human fetal RPE cells at Passage 4 were used for research and the medium was changed every 2-3 days. Cell division was inhibited by treatment with 10 *μ*g/mL mitomycin C (Wako) for 6 hours. Cultured cells were recorded using a laser scanning confocal microscope (IX81; Olympus, Tokyo, Japan).

### 2.2. Cytotoxicity Assay

The cell damage rate was measured by performing a lactate dehydrogenase (LDH) cytotoxicity test (Wako) following the manufacturer's instructions. ROCK inhibitor Y27632 was purchased from Wako. RPE cells in preconfluent medium were seeded at a density of 1.0 × 10^5^ cells/cm^2^ on 96-well plates, and preconfluent medium was switched to postconfluent medium for 2 weeks after confluence was reached. RPE cells were cultured in postconfluent medium with four different dilutions of Y27632 (0, 10, 100, and 1000 *μ*M) for 24 hours, and culture supernatants were evaluated at 1 day after Y27632 administration (each *n*=5). The absorbance of each supernatant treated with a chemiluminescent reagent was recorded using a multimode microplate reader (Varioskan®; Thermo Scientific), and the cell damage rate was calculated with the following equation: cell damage rate (%) = ((sample absorbance) − (negative control absorbance))/((positive control absorbance) − (negative control absorbance)) × 100. The positive control was RPE supernatant treated with 0.2% Tween 20 for 45 min at 37°C.

### 2.3. Number of Viable Cells

The number of viable cells was quantified using the CellTiter-Glo® 2.0 Assay (Promega, Madison, WI, USA), following the manufacturer's instructions. In cell adhesion assays, RPE cells in preconfluent medium with and without 10 *μ*M Y27632 were seeded at a density of 1.0 × 10^4^ cells/cm^2^ on 96-well plates, and the number of viable cells was measured at 2 and 24 hours after seeding (each *n*=5). In cell proliferation assays, RPE cells in Y27632-free preconfluent medium were seeded (at 1.0 × 10^4^ cells/cm^2^) in 96-well plates, and at 24 hours after seeding, the culture medium was changed to preconfluent medium with and without 10 *μ*M Y27632; cell viability was measured at 2, 3, and 7 days after seeding (each *n*=5). At each time point, cells were washed twice with PBS and cultured in Y27632-free preconfluent medium at room temperature for 30 minutes, and then equal volumes of a chemiluminescent reagent were added. The luminescence in each well was recorded using a multimode microplate reader (Varioskan®) and standardized to the luminescence of the control.

### 2.4. Immunofluorescence Assays

The methods used for RPE immunocytochemistry have been described previously [20]. F-actin was stained with Alexa-Fluor™ 594 phalloidin (1/40, Thermo Fisher Scientific). Vinculin was detected with a primary antibody (rabbit, 1/50; Thermo Fisher Scientific). Bound primary antibodies were detected with Alexa Fluor 488-labeled goat anti-rabbit IgG secondary antibodies (1/500, Invitrogen), and nuclei were stained with 4′,6-diamidino-2-phenylindole (DAPI) (1 *μ*g/ml; Molecular Probes, Thermo Fisher Scientific). In Hematoxylin-Eosin staining, porcine RPE-choroid-scleral fragments were fixed (SuperFix®, Kurabo, Osaka, Japan), dehydrated, embedded in paraffin, and sectioned at 12 *μ*m thickness using a microtome. The deparaffinized sections were hydrated with graded ethanol series, stained with Meyer's hematoxylin and eosin, dehydrated with graded ethanol series, and cleared by xylene before mounting. Samples were imaged using a laser scanning confocal microscope (LSM-700; Zeiss, Oberkochen, Germany). Cell spreading area and FA area were quantified using Fiji imaging software (ImageJ, NIH) from confocal images of phalloidin- or vinculin-stained RPE cells treated with and without 10 *μ*M Y27632. Nonoverlapping RPE cells were selected manually. Cell surface area was calculated by using F-actin-staining area parameter processed using particle analysis (analyze particles command) after image thresholding based on F-actin signal (remove outliers command), size exclusion of noise pixels (subtract background command), and image binarization (make binary command) [21, 22] (each *n* ≥ 50). FA area was similarly extracted and calculated from vinculin staining areas (each *n* = 10).

### 2.5. Fluorescence-Activated Cell Sorting

RPE cells treated with and without 10 *μ*M Y27632 were washed twice with PBS and dissociated using Accutase (Funakoshi, Tokyo, Japan) for 15 minutes; the collected floating and adherent cells were resuspended, at 1.0 × 10^5^ cells/500 *μ*L, in PBS containing 2% FBS (each *n* = 5) and then incubated with Annexin V-FITC (Annexin V-FITC Kit System for Detection of Apoptosis; Beckman Coulter, Brea, CA, USA) for 15 minutes at room temperature in the dark and stained by 1 mg/mL propidium iodide (PI; 1 : 1000, Dojindo, Kumamoto, Japan) before assay. For cell cycle synchronization, RPE cells were cultured with 2.5 mM thymidine for 24 hours; synchronized cells were washed twice with PBS, cultured in the thymidine-free medium, and dissociated using 0.25% trypsin-EDTA 2, 4, 6, 8, 12, 16, 24, and 36 hours after block release. Lastly, cells were fixed in ethanol (overnight, −20°C) and incubated with RNase (30 minutes, 37°C) and then PI (10 minutes, 4°C). Stained RPE cells were passed through a cell strainer (BD, Franklin Lakes, NJ, USA), and cell profiles were analyzed on a FACSCanto™ II flow cytometer (BD). The data were analyzed using FlowJo software (FlowJo, Ashland, OR, USA).

### 2.6. Wound Healing Assay

**Figure 1:**
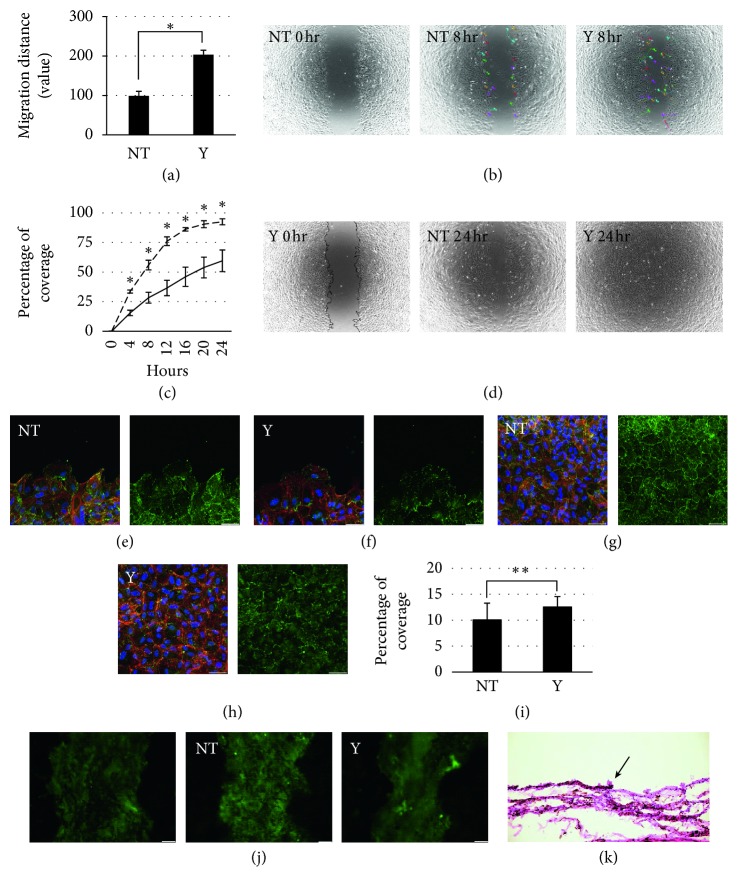
The effect of 10 *μ*M Y27632 on RPE wound healing. (a) Histogram showing cell migration distances of RPE cells treated with and without Y24632, *n*=80 for each; ^*∗*^ *P* < 0.01. (b) The left figure represents phase-contrast image of untreated RPE cells at 0 hours after Y27632 administration. The middle and right figures represent automated visual-tracking of RPE cells treated with (right) and without (middle) Y24632 at 8 hours after Y27632 administration, *n*=20 for each. (c) Time course showing cell coverage percentage of RPE cells treated with and without Y24632 at 0, 4, 8, 12, 16, 20, and 24 hours after Y27632 administration. The solid line represents untreated RPE cells. The dashed line represents Y27632-treated RPE cells, *n*=4 for each; ^*∗*^ *P* < 0.01. (d) The left figure represents the open wound field between cells in the imported images, which were manually outlined. The middle and right figures represent phase-contrast images of RPE cells treated with (right) and without (middle) Y24632 at 24 hours after Y27632 administration. (e) F-actin (red), vinculin (green), and DAPI (blue) stained confocal images of wound-adjacent untreated RPE cells. (f) F-actin (red), vinculin (green), and DAPI (blue) stained confocal images of wound-adjacent Y24632-treated RPE cells. (g) F-actin (red), vinculin (green), and DAPI (blue) stained confocal images of untreated RPE cells far from wound sites. (h) F-actin (red), vinculin (green), and DAPI (blue) stained confocal images of Y24632-treated RPE cells far from wound sites. (i) Histogram showing cell coverage percentage of the *ex vivo* porcine RPE cells treated with and without Y24632 at 24 hours after Y27632 administration, *n*=15 for each; ^*∗∗*^ *P* < 0.05. (j) The autofluorescence images of porcine RPE-choroid-scleral fragment. RPE cells blocked scleral autofluorescence, and the scraped RPE area is represented as a green area. The figures represent the autofluorescence images of porcine RPE-choroid-scleral fragment before Y27632 administration (left) and treated with (right) and without (middle) Y24632 at 24 hours after Y27632 administration. (k) Hematoxylin-Eosin stained image of porcine RPE-choroid-scleral fragment. Black arrow represents the wound edge, and scraped RPE area is on the right side of black arrow.

RPE cells in Y27632-free medium were seeded (at 1.0 × 10^5^ cells/cm^2^) in noncoated 24-well plates (CytoSelect™ 24-well Wound Healing Assay, Cosmo Bio, Tokyo, Japan), and 24 hours later, the wound healing plate inserts were gently removed. Next, RPE cells were cultured in preconfluent medium with and without 10 *μ*M Y27632, and time-lapse imaging (BZ-X700; Keyence, Osaka, Japan) was used to record cells every 30 minutes (each *n* = 4); imaging sequences were used to produce wound healing movies and were imported into digital imaging software (Adobe Photoshop CS2, Adobe Systems Inc., San Jose, CA, USA). We manually outlined open wound fields between the RPE cells in imported images (Figure 1(d)), quantified the pixels within the enclosed areas by using Photoshop's Info Palette, and calculated cell coverage percentage (%) as 100 − (open wound field pixel numbers at each time point)/(open wound field pixel numbers at 0 hour) × 100. We traced 10 cells at wound edge using a tracking tool (BZ-X700; Keyence) until 8 hours after Y27632 administration, at which point RPE cells reached the opposite wound edge. Cell migration distance was obtained by adding actual measurement value of all migration distances (each *n* = 80). Cells that divided were excluded from the analysis.

The porcine eyes were enucleated, made 3-4 holes with a 20 G needle, and placed in preconfluent medium. After transporting the porcine eyes to our laboratory, the porcine cornea, conjunctiva, iris, lens, vitreous, and sensory retina were removed. We then made 4 to 5 radial incisions from the edges to the equator of the retina to prepare RPE-choroid-scleral fragments. The porcine RPE cells were scraped by a silicone-tipped brush backflush needle, and the RPE-choroid-scleral fragments were transferred to the 12-well plates containing preconfluent medium with and without 10 *μ*M Y27632, and 24 hours later, the RPE-choroid-scleral fragments were fixed (SuperFix®; Kurabo) for wound healing assay and Hematoxylin-Eosin staining. We confirmed that scraping by the brush backflush needle removes only porcine RPE cells (Figure 1(k)).

### 2.7. Statistical Analysis

Values are expressed as means ± SEM; *P* < 0.05 was considered significant. Cell surface area, vinculin puncta area, number of viable cells, Annexin-V positive/PI negative rate, cell migration distance, and cell coverage rate were evaluated using the Mann–Whitney *U*-test. Cell damage rate and cell cycle phase ratio (G1, S, and G2/M) were analyzed by performing one-way analysis of variance (ANOVA) followed by Scheffe's test.

## 3. Results

### 3.1. Effect of Y27632 on RPE Cell Morphology

**Figure 2:**
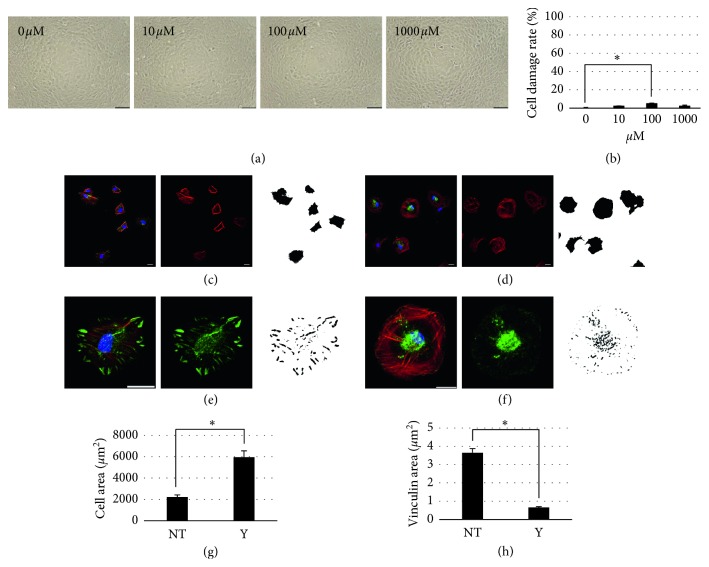
The effect of Y27632 on RPE cell toxicity, cell spreading, and focal adhesion size. (a) Phase-contrast images of RPE cells treated with four different dilutions of Y24632 (0, 10, 100, and 1000 *μ*M) at 1 day after Y27632 administration (scale bar, 50 *μ*m). (b) Histogram showing the cell damage rate of RPE cells treated with four different dilutions of Y27632 at 1 day after Y27632 administration, *n*=5 for each; ^*∗*^ *P* < 0.01. (c) F-actin (red), vinculin (green), and DAPI (blue) stained confocal images (left and middle figures) and binarized image (right figure) of untreated RPE cells. (d) F-actin (red), vinculin (green), and DAPI (blue) stained confocal images (left and middle figures) and binarized image (right figure) of Y27632-treated RPE cells. (e) F-actin (red), vinculin (green), and DAPI (blue) stained confocal images (left and middle figures) and binarized image (right figure) of untreated RPE cells. (f) F-actin (red), vinculin (green), and DAPI (blue) stained confocal images (left and middle figures) and binarized image (right figure) of Y27632-treated RPE cells. (g) Histogram showing the F-actin-based cell surface area of RPE cells treated with and without Y27632, *n* ≥ 50 for each; ^*∗*^ *P* < 0.01. (h) Histogram showing the vinculin size of RPE cells treated with and without Y27632, *n*=10 cells for each; ^*∗*^ *P* < 0.01.

At 10 *μ*M, Y27632 treatment exhibited no obvious RPE cytotoxicity in morphological and LDH assay (Figures 2(a) and 2(b)).

To elucidate the effect of Y27632 on actomyosin contractility of RPE cells, we seeded RPE cells in preconfluent medium with and without 10 *μ*M Y27632, and 2 hours later, we measured the RPE cell spreading area as the actin-based cell surface area. 10 *μ*M Y27632 substantially increased RPE cell spreading area by reducing actomyosin contractility (nontreatment: 2214 ± 203 *μ*m^2^; Y27632 treatment: 5961 ± 595 *μ*m^2^; Figures 2(c), 2(d), and 2(g)).

Y27632 effect on RPE cell FA was evaluated using a vinculin-based morphological assay. We seeded RPE cells in preconfluent medium with and without 10 *μ*M Y27632 and determined the mean size of vinculin puncta from immunofluorescence images acquired at 2 hours after seeding. 10 *μ*M Y27632 treatment decreased RPE cell FA size by reducing actomyosin contractility (nontreatment: 3.65 ± 0.23 *μ*m^2^; Y27632 treatment: 0.66 ± 0.04 *μ*m^2^; Figures 2(e), 2(f), and 2(h)).

### 3.2. Effect of Y27632 on the Number of RPE Cell Attachment

**Figure 3:**
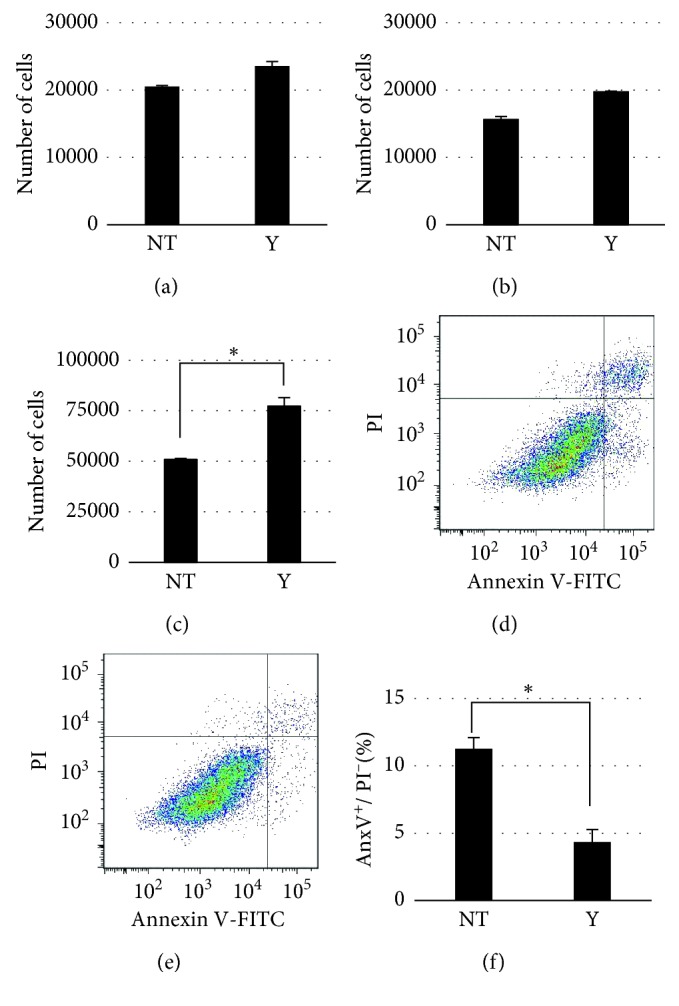
The effect of 10 *μ*M Y27632 on RPE cell attachment and apoptosis. (a) Histogram showing the number of viable RPE cells treated with and without Y24632 at 2 hours after seeding, *n*=5 for each. (b) Histogram showing the number of viable MMC-treated RPE cells treated with and without Y24632 at 2 hours after seeding, *n*=5  for each. (c) Histogram showing the number of viable RPE cells treated with and without Y24632 at 24 hours after seeding, *n*=5 for each; ^*∗*^ *P* < 0.01. (d) Flow-cytometry chart of Annexin V/PI of untreated RPE cells. (e) Flow-cytometry chart of Annexin V/PI of Y27632-treated RPE cells. (f) Histogram showing Annexin-V positive/PI negative rate of RPE cells treated with and without Y24632 at 24 hours after seeding, *n*=5 for each; ^*∗*^ *P* < 0.01.

Because Y27632 treatment decreased the size of puncta of vinculin 2 hours after Y27632 administration, which regulates cell adhesion strength, the number of attached cells was evaluated. The attached RPE cells treated with and without 10 *μ*M Y27632 were quantified using a luminescent cell viability assay. The number of viable untreated and 10 *μ*M Y27632-treated RPE cells at 2 hours after seeding was 20519 ± 152 and 23569 ± 673, respectively, with no significant differences (Figure 3(a)). Similar results were obtained with RPE cells treated with mitomycin C to exclude the influence of cell division (Figure 3(b)): viable cell numbers at 2 hours were, respectively, 14861 ± 596 for nontreatment and 18893 ± 292 cells for 10 *μ*M Y27632 treatment, with no significant differences. These results indicated that 10 *μ*M Y27632 treatment did not decrease the number of RPE cell attachment despite weakening RPE cell adhesion. Moreover, we measured the number of attached cells at 24 hr postseeding to clarify the influence on RPE cell adhesion by Y27632 administration. The number of viable untreated and 10 *μ*M Y27632-treated RPE cells at 24 hours after seeding was 50904 ± 557 and 77317 ± 4201, respectively (Figure 3(c)); therefore, 10 *μ*M Y27632 treatment markedly promoted the number of attached cells at 24 hours after seeding. Previously, increasing cell spreading [23] and inhibiting actomyosin hyperactivation [24] were shown to lead to cell survival by suppressing apoptosis; thus, we examined the effect of Y27632 on RPE cell apoptosis. RPE cells in preconfluent medium with and without 10 *μ*M Y27632 were seeded, and 24 hours later, floating and attached cells were quantified using Annexin-V/PI assay and flow cytometry; the obtained Annexin-V positive/PI negative rates were 11.3 ± 0.8% and 4.3 ± 0.9% for untreated and 10 *μ*M Y27632-treated RPE cells, respectively (Figures 3(d)–3(f)). Therefore, weakening RPE cell adhesion strength by 10 *μ*M Y27632 administration did not reduce the number of RPE cell attachment, but the treatment potently increased the number of attached cells by suppression of RPE cell apoptosis.

### 3.3. Effect of Y27632 on RPE Cell Motility during Wound Healing

We next investigated whether Y27632-induced vinculin downregulation promotes RPE cell motility during wound healing *in vitro*. After RPE cells reached confluence, we removed the inserts of 24-well wound healing assay plates, which left a 0.9 mm wide open wound field between cells (Figure 1(b), left figure); the culture medium was then changed to preconfluent medium with and without 10 *μ*M Y27632, and the cells were imaged every 30 minutes. To evaluate the Y27632 effect on the cell motility, we tracked cells at wound edges in time-lapse imaging to measure cell migration distances up to 8 hours after 10 *μ*M Y27632 administration, which were 98.9 ± 11.6 and 203.4 ± 11.2, respectively, for untreated and Y27632-treated RPE cells (Figures 1(a) and 1(b); Movies S1 and S2). To determine whether the increased cell motility enhanced wound healing, we measured the cell coverage percentage (cell-covered pixels in wound fields) every 4 hours (Figure 1(d), left figure). Coverage percentage increased over time with both untreated and Y27632-treated RPE cells (Figure 1(d); Movies S3 and S4), with the percentages for Y27632-treated RPE cells being higher than those for untreated RPE cells at all time points (Figure 1(c)). Here, by performing immunofluorescence imaging, we confirmed that Y27632 treatment downregulated vinculin in wound-adjacent RPE cells: 10 *μ*M Y27632 reduced vinculin expression at both cell-cell adherens junctions and cell-ECM adhesions near wounds (Figures 1(e) and 1(f)). Vinculin was also similarly downregulated in RPE cells that were far from wound sites (Figures 1(g) and 1(h)). We next investigated whether Y27632 enhances RPE wound healing *ex vivo*. After porcine eyes were enucleated, the porcine cornea, conjunctiva, iris, lens, vitreous, and sensory retina were removed; we then made 4 to 5 radial incisions to prepare RPE-choroid-scleral fragments. The porcine RPE cells were scraped by a silicone-tipped brush backflush needle (Figure 1(k)) and the RPE-choroid-scleral fragments were transferred to the 12-well plates containing preconfluent medium with and without 10 *μ*M Y27632. Y27632 effect on RPE cell coverage percentage was evaluated using scleral autofluorescence-based imaging. The mean cell coverage percentage from autofluorescence images were acquired at 24 hours after transfer, which were 10.0 ± 3.7 and 12.5 ± 2.3%, respectively, for untreated and Y27632-treated RPE cells (Figures 1(i) and 1(j)).

### 3.4. Effect of Y27632 on RPE Cell Proliferation

**Figure 4:**
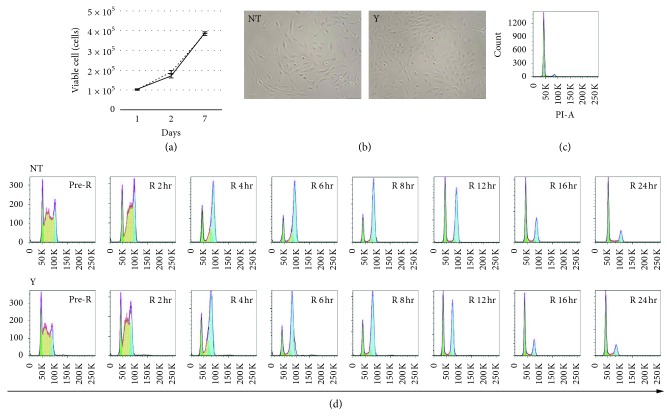
The effect of 10 *μ*M Y27632 on RPE cell proliferation. (a) Time course of the number of viable RPE cells treated with and without Y24632 at 2, 3, and 7 days after seeding. (b) Phase-contrast images of RPE cells treated with (right) and without (left) Y24632 at 3 days after seeding. (c) Flow-cytometry chart of the cell cycle distribution in G1, S, and, G2/M phase in untreated RPE cells. (d) Flow-cytometry chart of the cell cycle distribution in G1, S, and, G2/M phase in synchronized untreated (upper) and Y24632-treated (lower) RPE cells before and at 2, 4, 6, 8, 12, 16, 24, and 32 hours after block release.

ROCK is an effector of Rho GTPases which were previously shown to regulate cell cycle progression [25]. Cell proliferation plays a critical role in wound healing; therefore, we investigated whether Y27632 promotes RPE cell proliferation. RPE cells were seeded in Y27632-free preconfluent medium, and after 1 day, the medium was changed to control and 10 *μ*M Y27632-containing medium. The viable cell numbers were evaluated at 2, 3, and 7 days after seeding and were comparable between untreated and 10 *μ*M Y27632-treated RPE cells (Figures 4(a) and 4(b)). Moreover, we evaluated the cell cycle characteristics of synchronized RPE cells treated with and without 10 *μ*M Y27632. The cell cycle in RPE was synchronized by single-thymidine block technique, which was comparable with the double-thymidine block technique (data not shown). Flow-cytometry analysis revealed that untreated and 10 *μ*M Y27632-treated RPE cells were comparably distributed in distinct cell cycle phases before and at 2, 4, 6, 8, 12, 16, 24, and 32 hours after block release (Figure 4(d)); thus, 10 *μ*M Y27632 did not influence RPE cell proliferation.

## 4. Discussion

Anti-VEGF therapy has drastically improved prognosis for nAMD patients and is used as a first-line treatment. However, the detection of RPE tears, which develop spontaneously [1] or after laser photocoagulation [26], photodynamic therapy [27], and anti-VEGF therapy [28], has increased since anti-VEGF therapy introduction. Recently, most RPE tears have been reported as anti-VEGF therapy-associated complications, and a multicenter study reported a 16.8% incidence in 1280 eyes treated with anti-VEGF [29]; thus, RPE tears are no longer considered a rare complication of AMD. Photoreceptor viability and the retinoid cycle depend on adhesion with RPE, and RPE tears lead to morphological disruption of this essential structure. When RPE tears involve the fovea, sudden-onset central vision loss occurs in most patients. Although RPE tears not involving the fovea do not cause sudden central vision loss, visual prognosis is poor in the patients due to progressive scarring of the fibrovascular tissue [3]. No standard RPE tear therapy exists even though RPE tears cause severe vision loss; therefore, new therapeutic methods must be developed to counter the increasing RPE tear incidence. In the imaging of fundus autofluorescence derived from lipofuscin density in RPE, the area of RPE tear shows a markedly reduced autofluorescence signal; the hypoautofluorescent area features a high-contrast boundary that is accurately distinguishable in the early stage but becomes hazier and less demarcated in the late stage. A previous animal study showed that RPE cells at debridement-zone edges repopulate from the edge to the center [30]. Thus, RPE at RPE tear borders naturally proliferate and/or migrate to defective RPE areas, and we hypothesized that enhancing RPE cell migration might represent a useful therapeutic approach.

Cell migration mechanisms have been extensively studied, and Rho GTPases [31] play a pivotal role in regulating the biochemical pathways underlying cell migration. Rho, a member of the Ras GTPase superfamily, regulates actin cytoskeleton organization by activating downstream effectors that influence diverse aspects of cellular behavior, including cell morphology [32], motility [33], and polarity [34]. The Rho-associated kinases ROCK1 [35] (also known as p160ROCK [36]) and ROCK2 [35] are Rho GTPase effectors belonging to the AGC family of Ser/Thr kinases that play key roles in stress fiber formation by phosphorylating myosin light chain (MLC) and activating Lin11, Isl-1, and Mec-3 (LIM) kinase. MLC phosphorylation also activates myosin II ATPase, which results in stress fiber contraction. Stress fibers link FA proteins, including vinculin, and these proteins regulate cell adhesion strength through intracellular and extracellular mechanical tension. Cell adhesion strength is affected by vinculin-actin interaction: inhibition of stress fiber contractility by ROCK inhibitor treatment results in rapid vinculin release and accelerates cell migration by weakening cell adhesion strength. Our results showed that 10 *μ*M Y27632 inhibited actomyosin contractility, which led to cell spreading (Figure 2), reduction in vinculin area (Figure 2), and prolonged cell migration distance (Figure 1). Here, untreated RPE cells showed 50% wound coverage within 20 hours, but this coverage time was 40% of control (8 hours) after 10 *μ*M Y27632 treatment. Confocal images of vinculin staining showed that Y27632 treatment downregulated vinculin size at both cell-cell adherens junction and cell-ECM adhesions of wound-adjacent RPE cells (Figure 1). Vinculin downregulation at cell-cell adherens junction also helped RPE cell migration to wound areas. Previously, ROCK inhibitor treatment was shown to enhance wound healing in several ophthalmic cell types, including RPE, by promoting cell proliferation [15–18]. In contrast, we found that Y27632 did not enhance the RPE cell proliferation (Figure 4). Other studies have demonstrated that Rho GTPase regulation of cell cycle progression mostly involves G1/S transition [24] and that ROCK inhibitor suppressed proliferation in several cell types by blocking G1/S progression [37–39]. These findings do not contradict the enhancing effects of cell proliferation on wound healing, but instead demonstrate that scenarios exist in which the cell cycle effects do not apply because cell proliferation depends on cell status, culture conditions, and apoptosis suppression by ROCK inhibitor.

One limitation of this study is the efficacy of Y27632 *in vivo*; Y27632-induced RPE wound healing of *ex vivo* experiments was not improved as well as that of *in vitro* experiments. We showed that the coverage percentage of Y27632-treated RPE was 92.5% (control: 59.5%) at 24 hours *in vitro*, but this coverage percentage was 12.5% *ex vivo* (control: 10.0%). This difference can occur for the following reason: RPE cells *in vitro* migrated on dishes coated with laminin (CELLstart®), while RPE cells *ex vivo* migrated on Bruch membrane without RPE basement membrane obtained by brushing. The previous study compared RPE resurfacing on the RPE basement membrane and inner collagen layer in human submacular Bruch's membrane explants and decreased RPE resurfacing of inner collagen layer explants compared with RPE basement membrane explants [40]. The rolled up RPE cells in RPE tear patients contained RPE basement membrane [41], suggesting that Y27632 does not drastically improve RPE wound healing in the defected area *in vivo*. However, the porcine RPE cells used for the *ex vivo* experiments are from the cadaveric eyes, which are exposed to long-term hypoxic stress. The porcine eyes were acquired 2-3 hours after death and were placed in culture medium for 4 hours until RPE-choroid-scleral fragments were prepared. This condition could change the state of cells and decreased the efficacy of Y27632-induced RPE wound healing *ex vivo*. Another limitation of this study is the use of fetal RPE cells; AMD patients with RPE tears harbor a senescent retinal pigment epithelium. Here, 10 *μ*M Y27632 notably enhanced wound healing in fetal RPE cells. Future studies must investigate how 10 *μ*M Y27632 affects senescent RPE cells to assess the potential clinical application of this treatment. Regarding ROCK inhibitor safety, no severe systemic or local side effects were reported in clinical studies on eye drop treatments in healthy people and patients with corneal endothelial dysfunction [42]. Another ROCK inhibitor, ripasudil eye drop (K-115), is clinically applied to glaucoma [43]. The efficacy and safety of intravitreal injection of 10 *μ*M Y27632 must be evaluated in animal experiments.

## 5. Conclusions

We report here that ROCK signaling inhibition by 10 *μ*M Y27632 markedly promoted RPE cell motility, which resulted in enhanced wound healing. Thus, 10 *μ*M Y27632 holds considerable potential for use in RPE tear therapy. We hope that ROCK inhibitor administration will lead to a new treatment for AMD patients with RPE tears.

## Data Availability

Data are available from Hiroyuki Kamao (hironeri@med.kawasaki-m.ac.jp) for researchers who meet the criteria for access to confidential data.

## Supplementary Materials

Supplementary Materials Supplementary digital contents demonstrated the imaging of cell migration distance (Movie S1: NT; Movie S2: Y27632) and cell coverage percentage (Movie S3: NT; Movie S4: Y27632).

## References

[1] Hoskin A., Bird A. C., Sehmi K. (1981). Tears of detached retinal pigment epithelium. *British Journal of Ophthalmology*.

[2] Bressler N. M., Bressler S. B., Fine S. L. (1988). Age-related macular degeneration. *Survey of Ophthalmology*.

[3] Chuang E. L., Bird A. C. (1988). Repair after tears of the retinal pigment epithelium. *Eye*.

[4] Caramoy A., Kichhof B., Fauser A. (2011). Retinal pigment epithelium tears secondary to age-related macular degeneration. *Archives of Ophthalmology*.

[5] Caramoy A., Fauser S., Kirchhof B. (2012). Fundus autofluorescence and spectral-domain optical coherence tomography findings suggesting tissue remodelling in retinal pigment epithelium tear. *British Journal of Ophthalmology*.

[6] Mendis R., Lois N. (2014). Fundus autofluorescence in patients with retinal pigment epithelial (RPE) tears: an *in-vivo* evaluation of RPE resurfacing. *Graefe’s Archive for Clinical and Experimental Ophthalmology*.

[7] Mukai R., Sato T., Kishi S. (2015). Repair mechanism of retinal pigment epithelial tears in age-related macular degeneration. *Retina*.

[8] Heimes B., Farecki M.-L., Bartels S. (2016). Retinal pigment epithelial tear and anti-vascular endothelial growth factor therapy in exudative age-related macular degeneration. *Retina*.

[9] Geiger B. (1979). A 130K protein from chicken gizzard: its localization at the termini of microfilament bundles in cultured chicken cells. *Cell*.

[10] Carisey A., Tsang R., Greiner A. M. (2013). Vinculin regulates the recruitment and release of core focal adhesion proteins in a force-dependent manner. *Current Biology*.

[11] Calderwood D. A. (2004). Talin controls integrin activation. *Biochemical Society Transactions*.

[12] Carisey A., Ballestrem C. (2011). Vinculin, an adapter protein in control of cell adhesion signalling. *European Journal of Cell Biology*.

[13] Dumbauld D. W., Shin H., Gallant N. D., Michael K. E., Radhakrishna H., García A. J. (2010). Contractility modulates cell adhesion strengthening through focal adhesion kinase and assembly of vinculin-containing focal adhesions. *Journal of Cellular Physiology*.

[14] Jannie K. M., Ellerbroek S. M., Zhou D. W. (2015). Vinculin-dependent actin bundling regulates cell migration and traction forces. *Biochemical Journal*.

[15] Leung T., Manser E., Tan L., Lim L. (1995). A novel serine/threonine kinase binding the Ras-related RhoA GTPase which translocates the kinase to peripheral membranes. *Journal of Biological Chemistry*.

[16] Yin J., Yu F.-S. (2008). Rho kinases regulate corneal epithelial wound healing. *American Journal of Physiology-Cell Physiology*.

[17] Okumura N., Koizumi N., Ueno M. (2011). Enhancement of corneal endothelium wound healing by Rho-associated kinase (ROCK) inhibitor eye drops. *British Journal of Ophthalmology*.

[18] Koga T., Koga T., Awai M., Tsutsui J.-I., Yue B. Y. J. T., Tanihara H. (2006). Rho-associated protein kinase inhibitor, Y-27632, induces alterations in adhesion, contraction and motility in cultured human trabecular meshwork cells. *Experimental Eye Research*.

[19] Croze R. H., Thi W. J., Clegg D. O. (2016). ROCK inhibition promotes attachment, proliferation, and wound closure in human embryonic stem cell-derived retinal pigmented epithelium. *Translational Vision Science & Technology*.

[20] Kamao H., Mandai M., Wakamiya S. (2014). Objective evaluation of the degree of pigmentation in human induced pluripotent stem cell-derived RPE. *Investigative Ophthalmology & Visual Science*.

[21] Godbout C., Castella L. F., Smith E. A. (2013). The mechanical environment modulates intracellular calcium oscillation activities of myofibroblasts. *PLoS One*.

[22] Tsai W.-H. (1985). Moment-preserving thresolding: a new approach. *Computer Vision, Graphics, and Image Processing*.

[23] Chen C. S., Mrksich M., Huang S., Whitesides G. M., Ingber D. E. (1997). Geometric control of cell life and death. *Science*.

[24] Ohgushi M., Matsumura M., Eiraku M. (2010). Molecular pathway and cell state responsible for dissociation-induced apoptosis in human pluripotent stem cells. *Cell Stem Cell*.

[25] Yamamoto M., Marui N., Sakai T. (1993). ADP-ribosylation of the rhoA gene product by botulinum C3 exoenzyme causes Swiss 3T3 cells to accumulate in the G1 phase of the cell cycle. *Oncogene*.

[26] Donald J., Gass M. (1984). Retinal pigment epithelial rip during krypton red laser photocoagulation. *American Journal of Ophthalmology*.

[27] Gelisken F., Inhoffen W., Partsch M., Schneider U., Kreissig I. (2001). Retinal pigment epithelial tear after photodynamic therapy for choroidal neovascularization. *American Journal of Ophthalmology*.

[28] Meyer C. H., Mennel S., Schmidt J. C., Kroll P. (2006). Acute retinal pigment epithelial tear following intravitreal bevacizumab (Avastin) injection for occult choroidal neovascularisation secondary to age related macular degeneration. *British Journal of Ophthalmology*.

[29] Chan C. K., Abraham P., Meyer C. H. (2010). Optical coherence tomography-measured pigment epithelial detachment height as a predictor for retinal pigment epithelial tears associated with intravitreal bevacizumab injections. *Retina*.

[30] Leonard D. S., Zhang X. G., Panozzo G., Sugino I. K., Zarbin M. A. (1997). Clinicopathologic correlation of localized retinal pigment epithelium debridement. *Investigative Ophthalmology & Visual Science*.

[31] Ridley A. J., Hall A. (1992). The small GTP-binding protein rho regulates the assembly of focal adhesions and actin stress fibers in response to growth factors. *Cell*.

[32] Paterson H. F., Self A. J., Garrett M. D., Just I., Aktories K., Hall A. (1990). Microinjection of recombinant p21rho induces rapid changes in cell morphology. *Journal of Cell Biology*.

[33] Takaishi K., Sasaki T., Kato M. (1994). Involvement of Rho p21 small GTP-binding protein and its regulator in the HGF-induced cell motility. *Oncogene*.

[34] Nobes C. D., Hall A. (1999). Rho GTPases control polarity, protrusion, and adhesion during cell movement. *Journal of Cell Biology*.

[35] Nakagawa O., Fujisawa K., Ishizaki T., Saito Y., Nakao K., Narumiya S. (1996). ROCK-I and ROCK-II, two isoforms of Rho-associated coiled-coil forming protein serine/threonine kinase in mice. *FEBS Letters*.

[36] Ishizaki T., Maekawa M., Fujisawa K. (1996). The small GTP-binding protein Rho binds to and activates a 160 kDa Ser/Thr protein kinase homologous to myotonic dystrophy kinase. *EMBO Journal*.

[37] Zhao Z., Rivkees S. A. (2003). Rho-associated kinases play an essential role in cardiac morphogenesis and cardiomyocyte proliferation. *Developmental Dynamics*.

[38] Kamiyama M., Utsunomiya K., Taniguchi K. (2003). Contribution of Rho A and Rho kinase to platelet-derived growth factor-BB-induced proliferation of vascular smooth muscle cells. *Journal of Atherosclerosis and Thrombosis*.

[39] Chen J., Guerriero E., Lathrop K., SundarRaj N. (2008). Rho/ROCK signaling in regulation of corneal epithelial cell cycle progression. *Investigative Opthalmology & Visual Science*.

[40] Wang H., Ninomiya Y., Sugino I. K., Zarbin M. A. (2003). Retinal pigment epithelium wound healing in human bruch’s membrane explants. *Investigative Opthalmology & Visual Science*.

[41] Toth C. A., Pasquale A. C., Graichen D. F. (1995). Clinicopathologic correlation of spontaneous retinal pigment epithelial tears with choroidal neovascular membranes in agerelated macular degeneration. *Ophthalmology*.

[42] Okumura N., Koizumi N., Kay E. P. (2013). The ROCK inhibitor eye drop accelerates corneal endothelium wound healing. *Investigative Opthalmology & Visual Science*.

[43] Tanihara H., Inatani M., Honjo M., Tokushige H., Azuma J., Araie M. (2008). Intraocular pressure-lowering effects and safety of topical administration of a selective ROCK inhibitor, SNJ-1656, in healthy volunteers. *Archives of Ophthalmology*.

